# Regular Low-Dose Oral Metronidazole Is Associated With Fewer Vesicovaginal and Rectovaginal Fistulae in Recurrent Cervical Cancer: Results From a 10-Year Retrospective Cohort

**DOI:** 10.1200/JGO.19.00206

**Published:** 2019-09-03

**Authors:** Reena George, Thotampuri Shanthi Prasoona, Ramu Kandasamy, Thenmozhi Mani, Shakila Murali, Roja Rekha, Jayaprakash Muliyil

**Affiliations:** ^1^ Christian Medical College, Vellore, India

## Abstract

**PURPOSE:**

Anaerobic necrosis in cervical cancer can lead to malodor, fistulae, and treatment abandonment. In this retrospective cohort study, we examined the association between maintenance metronidazole and the incidence of malignant fistulae in recurrent cervical cancer.

**METHODS:**

We screened all cervical cancer records registered between 2007 and 2016 in the local palliative care database at Christian Medical College, Vellore, India. There were 208 eligible patients with post-treatment residual/recurrent pelvic disease. Among them, 76 had received oral maintenance metronidazole 200 mg once per day for 2 to 86 weeks (interquartile range, 4-16 weeks).

**RESULTS:**

Seventy-two patients developed at least one fistula. Forty-nine had vesicovaginal fistulae, 10 had rectovaginal fistuale, and 13 developed both types of fistulae. Patients on maintenance metronidazole had fewer fistulae (22.4% *v* 41.7%; *P* = .005), a longer median fistula-free survival (42.9 months *v* 14.1 months; *P* < .001), and a postrecurrence survival of 11.5 months versus 8.7 months (*P* = .112). We performed Cox multivariable proportional hazards regression analysis on the data from the subset of 146 patients observed until death. Bladder/rectal infiltration had a higher risk of fistula (HR, 5.24; *P* = .011), whereas distant metastases (HR, 2.46; *P* = .012) and Eastern Cooperative Oncology Group performance status greater than 1 (HR, 1.64; *P* = .008) were associated with a higher risk of death. Maintenance metronidazole was associated with a lower risk of fistula (hazard ratio [HR], 0.33; 95% CI, 0.16 to 0.67; P = .002) and a lower risk of death (HR, 0.56; 95% CI, 0.39 to 0.81; P = .002).

**CONCLUSION:**

Our data indicate that there is a significant inverse association between oral maintenance metronidazole and malignant fistulae in locally recurrent cervical cancer. The impact of this simple intervention on pelvic symptoms, fistulae, and survival should be evaluated in prospective studies.

## INTRODUCTION

It is estimated that one woman dies with cervical cancer every 2 minutes; 266,000 die each year.^1^ Many die with uncontrolled symptoms of advancing pelvic cancer: necrotic malodorous discharge or bleeding from the central tumor; pain and edema as the disease infiltrates nerves and lymphatics; and obstruction, fistulae, and incontinence caused by anteroposterior invasion of the bladder and rectum.^2,3^ Caregivers struggle with smell, incontinence, and flies—especially in small crowded huts without easy access to toilets or running water.^4,5^ Some guidelines recommend repeated packing of the vagina with vinegar, soda bicarbonate, or metronidazole solutions to reduce malodorous discharge,^6,7^ but there are few evidence-based interventions for pelvic symptoms and little ongoing research, despite the magnitude of suffering.^8^

**FIG 1:**
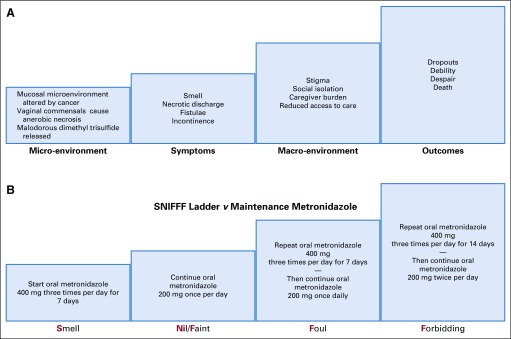
(A) Problems associated with uncontrolled anaerobic necrosis in advanced cervical cancer. (B) The Smell-Nill, Faint, Foul, or Forbidding (SNIFF) ladder versus maintenance metronidazole: initial and ongoing treatment with oral metronidazole titrated to severity of smell.

Metronidazole has been used to manage malodor in cancer since the 1980s.^9^ We reported that daily low-dose oral metronidazole seemed to provide more sustained malodor control than topical or intermittent use,^10^ and we suggested that dimethyl trisulfide, a malodorous product of fungatings cancers,^11^ could attract maggot-producing flies to patients with necrotic tissues.^12,13^ Therefore, we had proposed a Smell-Nill, Faint, Foul, or Forbidding (SNIFFF) ladder to clinically titrate prophylactic oral metronidazole according to the severity of smell,^10^ a strategy now recommended for resource-poor settings.^14^ Patients are given oral metronidazole 400 mg thrice per day for a week and then continue on 200 mg orally once per day. Breakthrough smell is treated with metronidazole 400 mg thrice per day for 1 or 2 weeks (Fig 1). With the increasing use of regular metronidazole, we observed that some of our patients with cervical cancer remained fistula free for long periods despite bulky rectal infiltration.

**CONTEXT**

**Key Objective**
Did women with recurrent cervical cancer who received maintenance metronidazole for malodor control develop fewer vesicovaginal and rectovaginal fistulae?
**Knowledge Generated**
Regular low-dose oral metronidazole was associated with a three-fold reduction in the risk of fistula. Maintenance metronidazole was also associated with a longer postrecurrence survival in the subset of patients observed until death.
**Relevance**
To our knowledge, this is the largest reported series of malignant vesicovaginal and rectovaginal fistulae. Our findings suggest that maintenance metronidazole has the potential to reduce anerobic necrosis-induced fistulae in advanced cervical cancer. The impact of metronidazole on pelvic symptoms, treatment completion, quality of life, and survival must be studied in randomized trials. If effective, this simple and inexpensive intervention can be readily translated into clinical practice, even in resource-limited settings.

Could maintenance metronidazole reduce fistulae in recurrent cervical cancer? We tested this hypothesis in our retrospective cohort.

## METHODS

### Setting

The Christian Medical College, Vellore, is a 2,500-bed, not-for-profit teaching hospital in South India. The palliative care unit is staffed by physicians trained in radiation oncology and palliative care, two oncology nurses, a social worker, a counselor, and a secretary. Ambulant patients are seen at least monthly in the outpatient clinic for symptom review, physical examination, titration of medications, counseling, and caregiver education. Follow-up care for nonambulant patients is done through a combination of clinic visits by the family caregiver, phone calls, and home visits for the most needy.

### Participants, Procedures, and Outcomes

**FIG 2:**
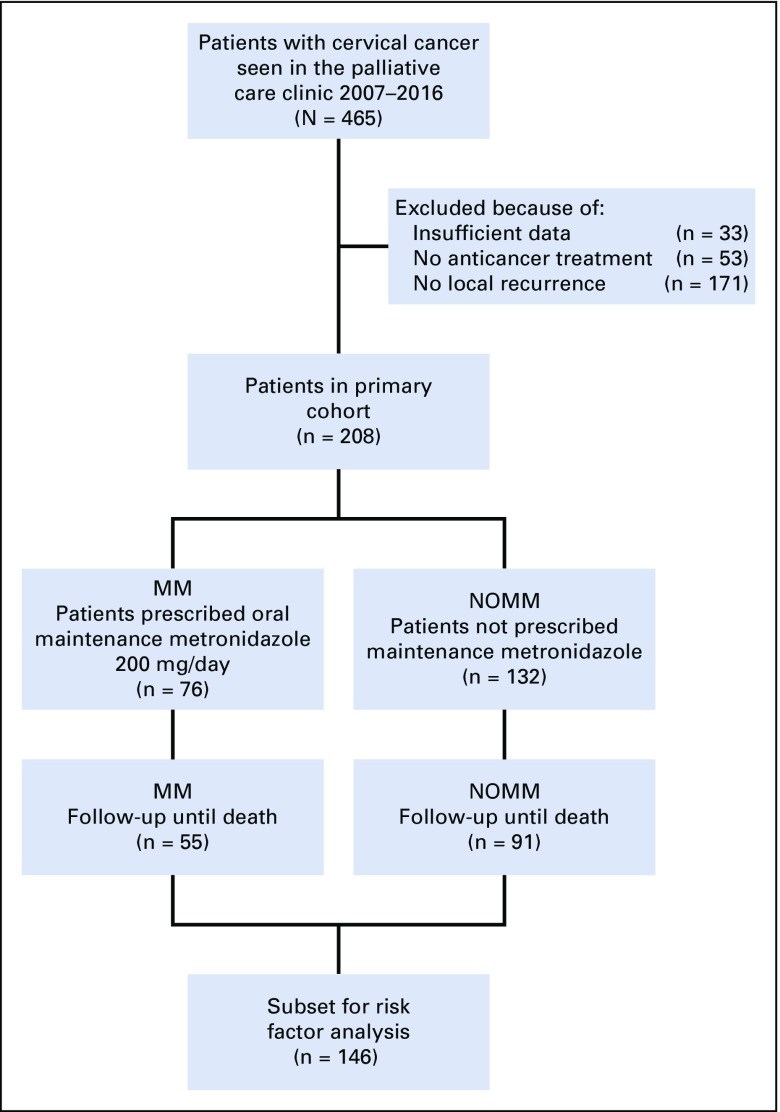
Flow diagram of patients considered for the study. MM, maintenance metronidazole; NOMM, no maintenance metronidazole.

We identified women with cervical carcinoma from our prospectively maintained database of patients who resided within 50 kilometers of the medical college. We included patients with recurrent or residual cervical cancer who attended the palliative care clinic between 2007 and 2016. We excluded patients who had not received anticancer treatment and those without pelvic recurrence. From our primary cohort, we also identified a subset of patients who had been on follow-up until death (Fig 2).

Data were obtained from prospectively maintained clinical and pharmacy records. Any patient who was prescribed metronidazole 200 mg once per day in tablet form on two consecutive clinic visits was included in the maintenance-metronidazole (MM) group. All others were in the no–maintenance-metronidazole (NOMM) group. Intermittent therapeutic courses of metronidazole were permissible in both groups. Data were extracted for number, type, and time to fistulae as well as for oncologic status, medication use, symptoms, clinical course, social circumstances, and date of death. For time-to-event outcomes, we defined local recurrence as the documentation of the following: gross disease on pelvic examination or biopsy confirmation of suggestive local disease. We defined fistula when a physician recorded either a visible or palpable defect in the vagina or the presence of urine or stool within the vaginal cavity with a coexistent history of leakage. The date of death was available in the clinical notes for two thirds of patients, as documented by telephone calls to the family by our social worker within a month when the patient missed a scheduled follow-up visit. Fistula and survival status were censored at the end of the study period (August 31, 2017) or at last follow-up. The study received approval from the institutional review board of the Christian Medical College, Vellore.

### Statistical Analysis

We compared clinicodemographic characteristics and fistula proportions across groups using χ^2^ and Fisher’s exact tests. The cumulative probabilities of fistula-free survival and postrecurrence survival were estimated by the Kaplan-Meier method. The log-rank test was used to compare survival curves. In the subset of patients who had been on follow-up until death with or without a fistula, Cox proportional hazards regression was performed to assess potential risk factors for fistula and survival. Multivariable Cox proportional hazards models were used to account for potential confounders. Because cross-sectional imaging was not available for most patients, covariables were limited to important clinicopathologic variables, such as stage, histology, treatment, and Eastern Cooperative Oncology Group performance status. The model assumption was verified by log –log S(t) plots and the global test. *P* values less than .05 were interpreted as statistically significant. All statistical analyses were performed in SPSS version 21.0 (SPSS, Chicago, IL).

## RESULTS

### Patient Characteristics

We identified 208 women who had locally recurrent cervical cancer and met study criteria. A subset of 146 had been observed until death (Fig 2). Only 31.2% of patients had received adequate primary treatment with radical radiotherapy/chemoradiotherapy followed by brachytherapy. Some patients had discontinued radical treatment; others had been suboptimally treated at various hospitals. Brachytherapy had not been given to 58.3% of patients; 9% had undergone a nonradical hysterectomy; and 1.4% were given chemotherapy without radiotherapy.

### Metronidazole Use and Outcomes

Seventy-six patients received between 2 to 86 weeks of maintenance metronidazole (median, 8 weeks; interquartile range, 4-16 weeks). The use of maintenance metronidazole increased from 12% in 2006 to 70% in 2015 to 2016. There were no other statistically significant baseline differences between the MM and NOMM groups (Table 1).

**TABLE 1:**
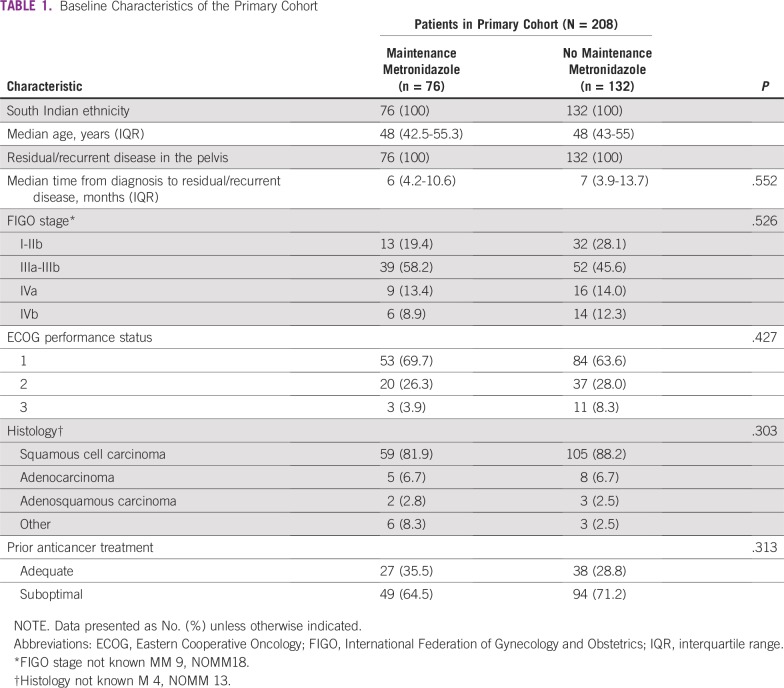
Baseline Characteristics of the Primary Cohort

**FIG 3:**
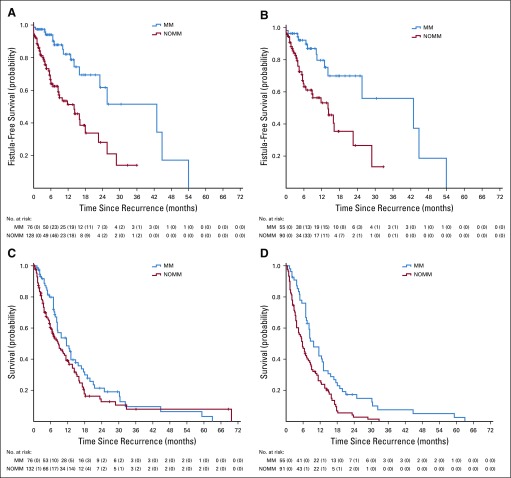
Kaplan-Meier curves by maintenance metronidazole group: (A) fistula-free survival in the primary cohort; (B) fistula-free survival in the subset observed until death; (C) postrecurrence survival in the primary cohort; and (D) postrecurrence survival in the subset observed until death. MM, maintenance metronidazole; NOMM, no maintenance metronidazole.

**FIG 4:**
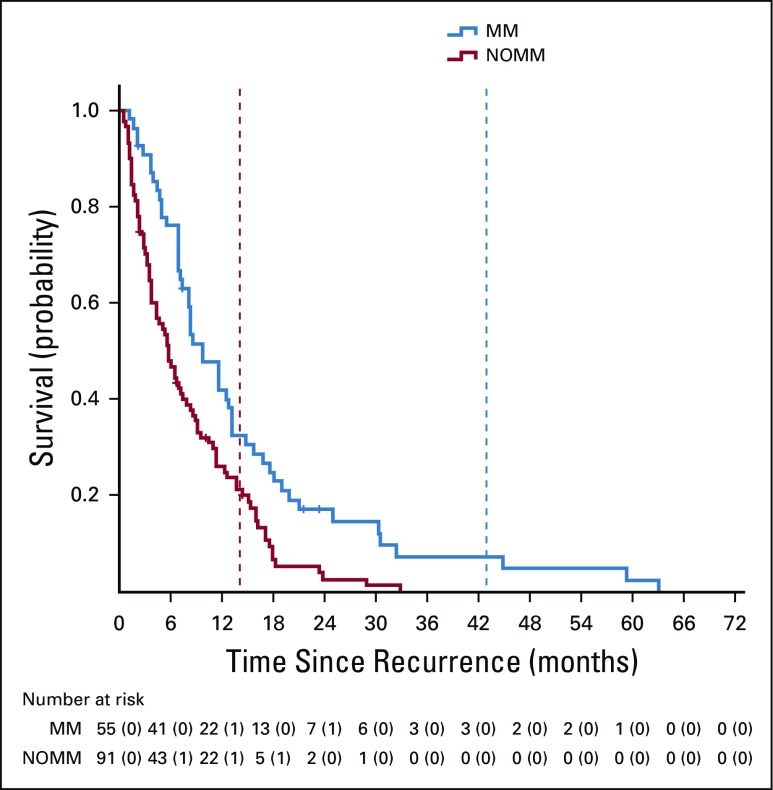
Kaplan Meier curves for postrecurrence survival. The superimposed vertical dotted lines indicate the time at which patients had a 50% risk of developing a fistula. MM, maintenance metronidazole; NOMM, no maintenance metronidazole.

Seventy-two patients (34.6%) developed at least one fistula; thirteen (6.2%) developed both vesicovaginal fistulae (VVF) and rectovaginal fistulae (RVF; Table 2). There were 62 patients with VVFs and 23 patients with RVFs in the entire cohort. The MM group had fewer fistulae than the NOMM group (22.4% *v* 41.7%; *P* = .005) and a significantly longer median fistula-free survival of 42.9 months (95% CI, 19.7 months to 66.2 months) versus 14.1 months (95% CI, 7.7 months to 20.4 months) in the NOMM group (*P* < .001; Fig 3). Because most patients died as a result of cancer within a year, the MM group was more likely to have been fistula free at death (77.6% *v* 58.3%; *P* = .005; Fig 4).

**TABLE 2:**
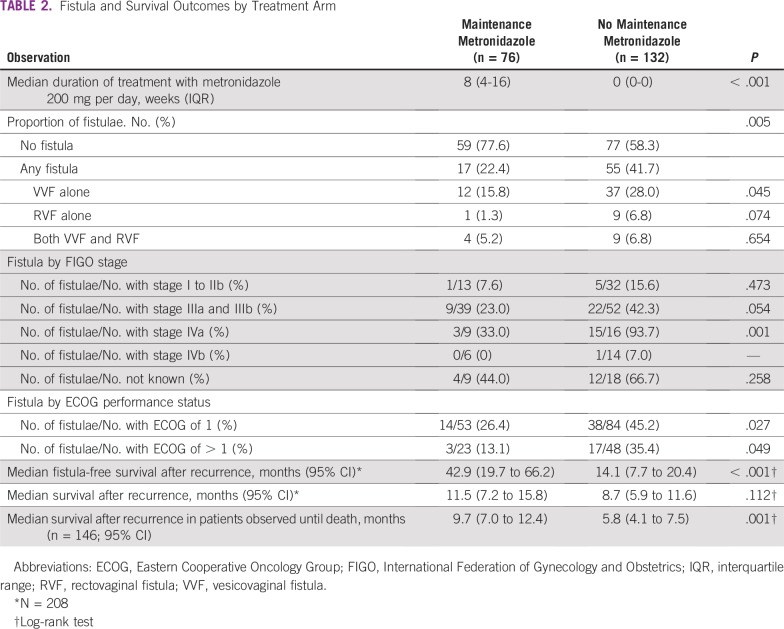
Fistula and Survival Outcomes by Treatment Arm

Sixty-two patients from the primary cohort were lost to follow-up and were censored by fistula and survival status at last visit. The median postrecurrence survival of the primary cohort was 11.5 months (95% CI, 7.2 months to 15.8 months) in the MM group versus 8.7 months (95% CI, 5.9 months to 11.6 months; *P* = .112) in the NOMM group. In the subset of 146 patients who were on follow-up until death, MM was associated with a significantly longer postrecurrence survival: 9.7 months (95% CI, 7 months to 12.4 months) versus 5.8 months (95% CI, 4.1 months to 7.5 months; *P* = .001; Table 2; Fig 3).

On multivariable Cox analysis in the subset who were on follow-up until death, the MM group had a lower risk of fistula (hazard ratio [HR], 0.33; 95% CI, 0.16 to 0.67; *P* = .002) and a lower risk of death (HR, 0.56; 95% CI, 0.39 to 0.81; *P* = .002; Tables 3 and 4). An increased risk of fistula was associated with infiltration of the bladder or rectum at diagnosis (HR, 5.24; 95% CI, 1.46 to 18.77; *P* = .011) and with unknown initial stage (Table 3). The group of patients with unknown initial stage included patients who were treated in other hospitals and presented to us with a recurrence without prior clinical records. Poor Eastern Cooperative Oncology Group performance status (HR, 1.64; 95% CI,1.14 to 2.35; *P* = .008) and stage IVB disease (HR, 2.46; 95% CI, 1.22 to 4.96; *P* = .012) were associated with an increased risk of death (Table 4).

**TABLE 3:**
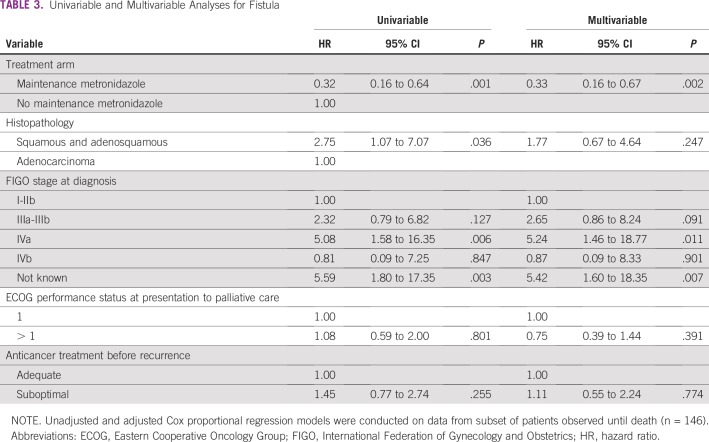
Univariable and Multivariable Analyses for Fistula

**TABLE 4:**
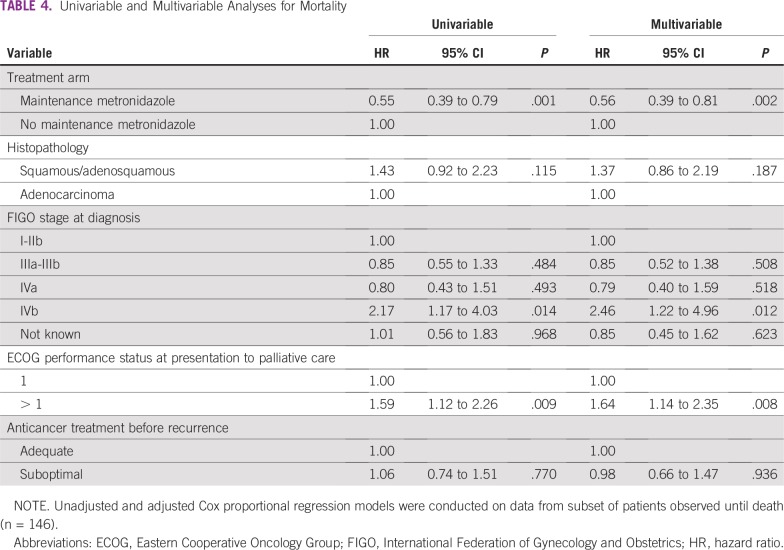
Univariable and Multivariable Analyses for Mortality

## DISCUSSION

To our knowledge, this is largest series of malignant VVF or RVF reported in recurrent cervical cancer. We observed that low-dose daily oral metronidazole was associated with a three-fold reduction in the risk of fistula and a possible improvement in survival.

First, we found that fistulae were common in our cohort. Prior studies reported VVF rates of 40% to 50% in recurrent stage IVA disease and 20% in nonrecurrent stage IVA cancer.^15,16^ Among our patients with stage IVA disease, 90% of the NOMM group and 33% of those in the MM group eventually developed a fistula. In the cohort as a whole, 41.7% of the NOMM group and 22.4% of the MM group developed fistulae. At a conservative estimate of 10%, more than 25,000 women with untreated or recurrent cancer could be dying with malignant fistulae each year. Yet, there is little in literature about the incidence, prevention, or management of this problem.

Nine tenths of the cervical cancer deaths occur in the developing world.^1^ High dropout rates are reported among patients with advanced cervical cancer.^3,17^ It is embarrassing for women who leak urine, stool, or smelly vaginal discharge to travel by public transport and wait in crowded cancer clinics. One adult diaper costs more than an elderly woman’s daily subsistence allowance. On palliative home visits, we have seen pelvic fistulae cause messy and lonely deaths. Caregivers withdraw, marriages break down, and some patients even develop maggots in the perineum. Thus isolated, patients stop coming for radiotherapy or palliative care.

Second, we noted that VVFs were more common than RVFs in our retrospective cohort. In a series of 67 patients with stage IVA disease, 10% of tumors infiltrated only the rectum, 10% infiltrated both bladder and rectum, and 80% infiltrated only the urinary bladder.^18^ The posterior bladder wall has a long plane of contact with the anterior wall of the vagina, and tumor necrosis could breach epithelial barriers to create a fistula. Sun et al^19^ reported that, if bladder wall involvement was longer than 1 inch, there was a five-fold increase in risk of VVF. In the same series, anterior tumor necrosis on magnetic resonance imaging was the greatest risk factor for a VVF (adjusted HR, 34.13; 95% CI, 4.07 to 286; *P* = .001).

The reduction of tumor necrosis within the septum is the likely reason for our third finding—a three-fold reduction in the risk of fistula in the group that received metronidazole. We had prescribed maintenance metronidazole for malodor and discharge caused by vaginal anaerobes, not specifically for fistula prophylaxis. Although metronidazole has not been reported for fistula prevention in cervical cancer, it has been beneficial in other fistulating conditions.

A 10-day course of metronidazole has reduced postlaryngectomy pharyngocutaneous fistulae in a blinded randomized trial.^20^ Longer courses of metronidazole have been used in Crohn’s disease.^21,22^ A single-arm study found that metronidazole 1,000 to 1,500 mg given for 3 to 36 months healed fistulae in half of the patients with refractory Crohn’s disease and reduced pain and discharge in the majority. Disulfiram-like reactions were not observed; metallic taste or paresthesias were reported only with full-dose metronidazole (20 mg/kg).^21,22^ Low-dose metronidazole for 3 months was associated with fewer recurrences of Crohn’s disease.^23^

In a randomized trial, postoperative metronidazole reduced the risk of perianal fistulae in patients with anorectal abscesses.^24^ A recent meta-analysis concluded that the administration of antibiotics was associated with significant reduction in the risk of fistula formation after anorectal abscess drainage.^25^

Fourth, the MM group had a median postrecurrence survival of 11.5 months, as compared to 8.7 months in the NOMM group. The latter is similar to previous palliative cohorts from India.^17^ The survival difference was significant only in the subset observed until death. Patients who received metronidazole developed fistulae later in the disease trajectory and, therefore, were likely to have remained continent, ambulant, and self-caring for longer (Fig 4). They may have been less vulnerable to the consequences of debility, chronic anaerobic inflammation, neglect, and therapeutic nihilism. Nevertheless, survival outcomes from our retrospective data could have been influenced by other confounders and should be interpreted with caution.

The limitations of this study include the high proportion of patients with inadequate treatment, the relatively small numbers, the losses to follow-up, and the absence of cross-sectional imaging to study anatomic risk factors. The cohort does not represent the entire spectrum of patients in our region. Patients with better prognoses might not have been referred from oncology to palliative care; undiagnosed and untreated patients would have died without coming to hospital. Within our cohort, we prioritized close follow-up for patients who were most clinically and socially disadvantaged, and that bias could be reflected in our results.

Despite these limitations, we obtained previously unreported real-world data about fistula patterns on the interface of oncology and palliative care. Many patients were seen at home and observed until death. We could document fistula rates in different stages of disease, not just in stage IVA cancer. We found that, with a small number of staff; without expensive investigations; and through careful history, examination, and follow-up, we could optimize the use of inexpensive medications. Metronidazole is on the list of essential drugs,^26^ and it is available even where palliative radiotherapy is not.^3^

Are our results generalizable? Currently, there are no evidence-based measures to prevent malignant VVFs and RVFs. Palliative radiotherapy does not control vaginal discharge in all patients.^17^ Systemic metronidazole has shown efficacy against perianal and pharyngo-cutaneous fistulae in randomized trials.^20,24^ Vaginal necrosis in cervical cancer is a risk factor for fistula,^19^ and the reduction of anaerobic necrosis is a biologically plausible explanation for the benefit observed in our cohort.

We therefore suggest that maintenance metronidazole should be considered in women with anaerobic malodor, necrotic discharge, bulky disease, and poor access to health care. It should be used in conjunction with pain management, drug subsidy, and caregiver support to optimize outcomes. Metronidazole might be less effective when there is little anaerobic necrosis, such as in patients with low-volume pelvic disease and in fistulae precipitated by biopsies^27^ or bevacizumab.^28^

We need pragmatic randomized trials stratified for high-, middle-, and low-resource settings to obtain more robust data about the effect of metronidazole on pelvic symptoms, inflammatory and metabolic markers, local control, quality of life, caregiver stress, and survival. The role of metronidazole in fistula prevention also could be tested in necrotizing head and neck, anorectal, and esophageal cancers.

It took us more than a decade to note the link between malodor, metronidazole, and fistulae in our single-center cohort. Palliative care research networks are uncommon, and poverty-stricken patients with advanced cancers in the developing world fall on the blind spot of academic medicine and clinical trials.^26^ The symptom burden is high,^29^ but long symptom checklists may prevent engagement with the patient’s greatest need. We suggest a simple screening tool in the vernacular that asks the health care worker in the hospital or community to record the two most difficult problems for the patient, to document interventions that were done and problems that have no satisfactory answer. A global registry that records and audits these data can draw early attention to refractory problems and emerging solutions. Palliative interventions then can be systematically tested and disseminated.

There is an 18-fold difference in cervical cancer survival rates across the globe.^30^ Good symptom control improves compliance with radiotherapy. Inexpensive and readily available, maintenance metronidazole could improve worthwhile outcomes in women who die premature, preventable, and sometimes horrific deaths as a result of cervical cancer.
